# 

**Published:** 1885-08

**Authors:** 


					﻿Vol. VI.AUGUST, 1885.	No. 8.
TZEZZE
indcnπuIcnlPraditioncr
A MONTHLY
DEVOTED TO
DENTAL AND ORAL SCIENCE.
W. C. BARRETT, M. D., D. D. S.,
EDITOR.
The Hew York Dental Journal Association,
PUBLISHERS,
No. 35 West <Lθth Street, New York.
AND
11 NV. Chippewa St., Buffalo, N.Y.
$2.50 Per Annum in Advance, .... Single Copies, 25 Cents.
Entered at the Post-Office, Buffalo, N. Y., as Second-Class matter.
				

